# Conditional deletion of Neurexin-2 alters neuronal network activity in hippocampal circuitries and leads to spontaneous seizures

**DOI:** 10.1038/s41398-023-02394-6

**Published:** 2023-03-20

**Authors:** Mulatwa T. Haile, Sheraz Khoja, Gregory de Carvalho, Robert F. Hunt, Lulu Y. Chen

**Affiliations:** Department of Anatomy & Neurobiology, School of Medicine, University of California, Irvine, CA 92697 USA

**Keywords:** Molecular neuroscience, Hippocampus, Autism spectrum disorders

## Abstract

Neurexins (Nrxns) have been extensively studied for their role in synapse organization and have been linked to many neuropsychiatric disorders, including autism spectrum disorder (ASD), and epilepsy. However, no studies have provided direct evidence that Nrxns may be the key regulator in the shared pathogenesis of these conditions largely due to complexities among Nrxns and their non-canonical functions in different synapses. Recent studies identified *NRXN2* mutations in ASD and epilepsy, but little is known about Nrxn2’s role in a circuit-specific manner. Here, we report that conditional deletion of *Nrxn2* from the hippocampus and cortex (*Nrxn2* cKO) results in behavioral abnormalities, including reduced social preference and increased nestlet shredding behavior. Electrophysiological recordings identified an overall increase in hippocampal CA3→CA1 network activity in *Nrxn2* cKO mice. Using intracranial electroencephalogram recordings, we observed unprovoked spontaneous reoccurring electrographic and behavioral seizures in *Nrxn2* cKO mice. This study provides the first evidence that conditional deletion of *Nrxn2* induces increased network activity that manifests into spontaneous recurrent seizures and behavioral impairments.

## Introduction

Neurexins (Nrxns) are cell-adhesion molecules that have been consistently demonstrated to play critical roles in diverse synaptic functions including synapse assembly [1–3], presynaptic release machinery [2–8], and postsynaptic receptor signaling [2, 5, 6, 9]. The mammalian genome contains three *Nrxn* genes (*Nrxn1-3* in mice and *NRXN1-3* in humans); each *Nrxn* gene exists in long α- and short β-isoforms [10, 11] while *Nrxn1* has an additional γ-isoform [12]. The first study using conditional approach deleting all *Nrxn1,2,3αβ* in different brain regions discovered dramatically different phenotypes in glutamatergic and/or GABAergic synapses [6]. This signifies the multi-faceted role of Nrxns and the necessity to investigate Nrxns in a circuit-specific manner.

Genetic truncations and variations in *NRXN2* have been reported in human patients diagnosed with neuropsychiatric disorders including autism spectrum disorder (ASD) and epilepsy [13–16]. To date, 118 *NRXN2* mutations have been reported in humans (Supplementary Table 1); however, *Nrxn2* is understudied. Previous studies have examined ASD-related behaviors using constitutive *Nrxn2* knockout (KO) mouse models [17–19]. Specifically, the study by Born et al (2015) reported that germline *Nrxn2* deletion exclusively induced changes in excitatory, but not inhibitory, neurotransmission [17]. However, recent discoveries show that Nrxns have distinct circuit-specific function [5, 6] and *Nrxn2* is differentially expressed across brain regions in neuronal and non-neuronal cells [20, 21], suggesting a potential multifaceted regulatory role of *Nrxn2* in distinct circuits. A recent study using a conditional *Nrxn2* mouse model discovered that Nrxn2 has an inhibitory role that restricts excitatory synapses in the hippocampus [22]. Here, we conditionally deleted *Nrxn2* under the *Emx1Cre* promoter which is predominately expressed in excitatory neurons of neocortex and hippocampus [23, 24] regions that are heavily implicated in ASD and epilepsy [13–15]. *Emx1Cre* is also expressed, at lower levels or in a subset of cells, in other brain regions (i.e., striatum and olfactory bulb) and non-neuronal cells [25, 26] (Supplementary Figure 1; Supplementary Table 2). Overall, our data provides a novel conditional approach to investigate the role of *Nrxn2* on social and repetitive behaviors, susceptibility to spontaneous reoccurring seizures, and underlying hippocampal network activity.

## Materials and methods

### Animals

**Fig. 1:**
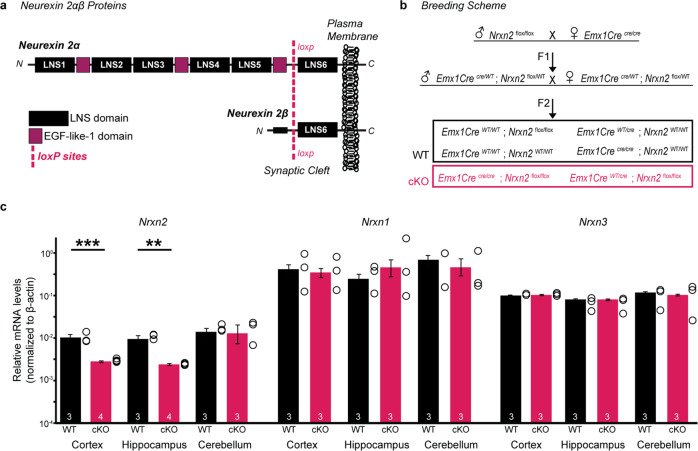
*Emx1Cre* driven deletion of *Nrxn2* in mouse brain. **a** Diagram illustrating α- and β- domain structures of Neurexin 2 (*Nrxn2*) (LNS = laminin/neurexin/sex-hormone binding globulin domain; EGF = epidermal growth factor-like 1 domain). The first exon shared by *Nrxn2* α- and β is flanked by loxP sites (*Nrxn2*^*flox/flox*^ mice). **b** Breeding scheme for conditional deletion of *Nrxn2* (*Nrxn2* cKO) by crossing *Nrxn2*^*flox/flox*^ mice with *Emx1*^*cre/cre*^ mice. The first filial (F1) generation results in heterozygous offspring, to generate the second filial generation (F2). F2 WT and *Nrxn2* cKO genotypes are listed: 18.75% of offspring were *Nrxn2* cKO while 43.75% were WT. Refer to supplementary Fig 1 and supplementary table 2 for expression levels of *Nrxn2* and *Emx1Cre* across brain regions. **c** mRNA measurements by qRT-PCR demonstrated that Cre recombinase reduced *Nrxn2* expression without altering *Nrxn1* or *Nrxn3* expression. Summary graphs of relative mRNA levels (normalized to actin) from cortex, hippocampus, and cerebellum. Refer to supplemental Fig1c for qRT-PCR probe alignments. Number of mice is listed inside the bar graphs. Each open circle in the summary graphs represents each mouse. Data is represented as means ± SEM from 3 WT and 4 *Nrxn2* cKO for *Nrxn2* (c); 3 WT and 3 *Nrxn2* cKO for *Nrxn1* and *Nrxn3* (**c**). Statistical assessments were performed by two-tailed Student’s *t*-test by comparing *Nrxn2* cKO to WT mice with **p < 0.01, ***p < 0.001.

Mice were maintained on a C57BL/6 J background and obtained from Jackson laboratories, *Nrxn2* mice (*Nrxn2*^*f/f*^) containing loxP-sites flanking exon 18 of the *Nrxn2* gene (stock # 031094) [6, 22] were bred with homozygous *Emx1*^*cre/cre*^ mice (stock # 005628) (Fig. 1). DNA was amplified using primers for *Nrxn2* (forward 5'-CAG GGT AGG GTG TGG AAT GAG GTC-3', reverse 5'-GTT GAG CCT CAC ATC CCA TTT GTC T-3') and *Emx1* (shared forward 5'-CAA CGG GGA GGA CAT TGA-3', WT reverse 5'-CAA AGA CAG AGA CAT GGA GAG C-3', Cre reverse 5'-TCG ATA AGC CAG GGG TTC3'). Resulting amplicons: 200-bp *Nrxn2*^*WT/WT*^, 350-bp *Nrxn2*^*f/f*^, 315-bp *Emx1*^*WT/WT*^, and 195 bp *Emx1*^*cre/cre*^. Age- and sex-matched WT and *Nrxn2* cKO littermates were used (Fig. 1b). Group housed mice were maintained on a 12/12-h light/dark cycle with food and water ad libitum. Sample sizes were determined based on literature values used for qRT-PCR, behavioral, rapid Golgi staining, electrophysiology, spontaneous and chemically induced seizure analyses. Mice were not randomized into groups since all the mice were subjected to the same treatment conditions for all experiments irrespective of their genotype. All experiments were performed blindly without the knowledge of the experimenter of mouse genotypes for all experiments. All procedures were conducted in accordance with the National Institute of Health Guide for the Care and Use of Laboratory Animals and protocols were approved by the Institutional Animal Care and Use Committee, University of California Irvine, including efforts to minimize suffering and animals used.

### *qRT-PCR*

RNA isolated (RNeasy minikit; Qiagen) from selected brain regions of male and female WT (*n* = 3 biological replicates with 2–3 technical replicates) and *Nrxn2* cKO (*n* = 4 biological replicates for *Nrxn*2, *n* = 3 biological replicates for *Nrxn*1 and *Nrxn*3 with 2–3 technical replicates for each) mice. Isolated RNAs (1 μg) were subjected to target-specific reverse-transcription for 1 h at 50 °C (Applied Biosystems). Pre-amplified cDNAs were then processed for RT-PCR analysis on LightCycler96 system (Roche Life Science). FAM-dye coupled detection assays were purchased from Integrated DNA Technologies. Primers and probes were designed as previously described [27].

Transcript levels were normalized to β-actin.

*Nrxn1*: forward: 5'-ACTACATCAGTAACTCAGCACAG-3'

reverse: 5'-ACAAGTGTCCGTTTCAAATCTTG-3'

probe: 5'-CTTCTCCTTGACCACAGCCCCAT-3'

*Nrxn2*: forward: 5'-GTCAGCAACAACTTCATGGG-3'

reverse: 5'-AGCCACATCCTCACAACG-3'

probe: 5'-CTTCATCTTCGGGTCCCCTTCCT-3'

*Nrxn3*: forward: 5'-GGGAGAACCTGCGAAAGAG-3'

reverse: 5'-ATGAAGCGGAAGGACACATC-3'

probe: 5'-CTGCCGTCATAGCTCAGGATAGATGC-3'

### Behavioral testing

Male and female mice were P90-P180 for the entire duration of behavioral studies and were handled for 5 days prior to all behavioral testing. Behavior paradigms were video recorded and analyzed (ANY-maze video tracking system, Stoelting Co) and scored by blinded investigators. Each task was replicated in the laboratory with at least two separate cohorts of mice.

#### Three-chamber social approach

It was assessed as described previously [28]. Prior to testing, age- and sex-matched (WT *n* = 10, cKO *n* = 13) unfamiliar “peer” mouse was habituated in an inverted wire cup (2 sessions, 15 min). The test mouse was first habituated to the center chamber with closed doors (10 min), then habituated to all three chambers without cues (10 min). Placement of cues (social cue: novel peer mouse confined in a cup, non-social cue: a novel empty cup) was counterbalanced (left or right chamber) with open doors allowing the test mouse access to both side chambers for 10 min. Time spent interacting with either cue was measured and calculated for peer/object ratio.

#### Olfactory habituation/dishabituation

Mice (WT *n* = 15, cKO *n* = 14) were placed in a clean mouse cage and allowed to acclimate for 30 min with an odorless cotton swab. Mice were then sequentially presented with cotton swabs saturated with water, two non-social odors (almond extract or coffee, 1:100), and two social odors (obtained from two sex-matched stranger cages). Each odor was presented three times (2 min each) with a 1 min intersession interval and sniffing time measured [29].

#### Nestlet-shredding

Mice (WT *n* = 15, cKO *n* = 14) were transferred to a new cage containing a single preweighed nestlet for 30 min. The non-shredded portion of the nestlet was weighed 24 h later to calculate the percentage of nestlet shredded [30].

#### Marble burying

Mice (WT *n* = 12, cKO *n* = 13) were placed in a rat cage (26 cm × 48 cm × 20 cm) containing unscented bedding (5 cm depth) and 20 marbles (15 mm, 5.2 g, distributed evenly) for 30 min. Marbles were considered buried if more than two-thirds of their surface is buried underneath the bedding [30].

#### Jumping bouts, digging, and self-grooming

These were tested for a duration of 10 min. Jumping bouts (WT *n* = 10, cKO *n* = 11) were defined as total jumping events (e.g., both hind legs are off the ground). Time spent in digging (WT *n* = 10, cKO *n* = 8) and in self-grooming (WT *n* = 11, cKO *n* = 12) (i.e., stroking or licking) were measured as described previously [31].

#### Open field

Mice (WT *n* = 14, cKO *n* = 15) were placed in the center of an opaque acrylic apparatus (30 cm × 23 cm × 23 cm) and allowed to explore for 5 min. Time spent in the center and the periphery zones, the total distance traveled, and velocity were measured [32].

#### Elevated plus maze

An elevated opaque acrylic maze (40 cm elevation) with four arms (30 cm long, 5 cm wide). Two opposite arms lack walls (open arms), and two opposite arms contain 15.25 cm high walls (closed arms). Mice (WT *n* = 8, cKO *n* = 9) were placed at the center facing the open arm and explored for 5 min. Total time spent and entries in each arm were measured [33].

#### Light/dark transition

A rectangular opaque acrylic apparatus (42 cm × 21 cm × 25 cm) divided into two compartments (2/3 light,1/3 dark) connected through an opening (3 cm high, cm wide). Mice (WT *n* = 15, cKO *n* = 13) were first placed in the light compartment and allowed to explore for 10 min. Total time spent in each compartment and the number of transitions were measured [34].

#### Object recognition memory

Task consisted of habituation, training, and testing phases in an opaque acrylic apparatus (30 cm × 23 cm × 23 cm). Habituation: 5 min, 3 days. Training: mice (WT *n* = 7, cKO *n* = 9) were placed in the center facing the two identical objects and explored for 10 min. Testing: 24 h post-training, one of the objects was replaced with a novel object (different shape and texture), and mice explored for 10 min. The location and the objects used were counterbalanced. Time spent exploring the objects was measured to calculate a discrimination index (DI = [time exploring novel-time exploring familiar]/ [time novel + time familiar] x 100) [32].

### Rapid Golgi staining and analysis

Male and female mice (P90-180) (WT *n* = 3 biological replicates with 3–4 technical replicates, cKO *n* = 3 biological replicates for cortical layer analysis, *n* = 4 biological replicates for dendritic branching and spine analysis with 3–4 technical replicates for each) were deeply anesthetized with isoflurane and quickly decapitated. Brains were collected and processed with Rapid GolgiStain kit (FD NeuroTechnologies), then cryoprotected and sectioned coronally (100 μm). Z-stack images (25 μm with 0.5 µm step-size) containing cortex and dorsal hippocampus at Bregma −2.06 mm (AP: −1.9 to −2.3, ML: 1.5 to 2.5 DV: 0.25 to 1.5) (Fig. 4a) were collected using a Keyence microscope (BZ-X800). Cortical layer analysis: length of each cortical layer was measured by ImageJ (NIH). An average of 34 images (10X) per mouse were analyzed. Dendritic branching and spine analyses: apical dendrites of CA1 pyramidal neurons in stratum radiatum were imaged at 60X. Dendritic branching (3–4 neurons per mouse) was determined by the number of dendritic intersections at each concentric circle and measured by Sholl analysis (ImageJ) [35]. For spine analysis, z-stack 60X images (three secondary dendrites per mouse) were first compiled in ImageJ and then imported to Reconstruct (SynapseWeb). Total spines on secondary apical dendrites were counted and spine head width was measured using the ‘Draw Line’ tool to determine spine maturity (spine head diameter ≥0.6 µm (mature) or <0.6 µm (immature)) [36].

### Electrophysiology

#### Brain slice preparation

Acute semi-horizontal hippocampal slices (300 μm) [37] were obtained from male and female WT and *Nrxn2* cKO mice (P90-180) (WT *n* = 4 for mEPSC, *n* = 3 for sEPSC, AMPA/NMDA ratio, and PPR, cKO *n* = 4 for mEPSC, sEPSC, and AMPA/NMDA ratio, *n* = 3 for PPR, with 3–5 technical replicates for each) using a vibratome (Leica V1200S) and brains were submerged in ice-cold N-Methyl D-glucamine cutting solution [38]. Slices were equilibrated (30 min, 31 °C) in aCSF containing (in mM): 119 NaCl, 26 NaHCO_3_, 1 NaH_2_PO_4_, 2.5 KCl, 11 glucose, 10 sucrose, 1.3 MgSO_4_-7 H2O, and 2.5 CaCl_2_ (290–295 mOsm, pH 7.4) (45 min, RT). Slices used for recordings contained dorsal hippocampus (Bregma: AP = −2 to −3; ML = ± 1.5 to 3; DV = 1.5 to 2.7) (Fig. 5a).

#### Whole-cell patch-clamp recordings

Recordings were conducted as previously described [6]. Briefly, recordings were obtained from CA1 pyramidal cells in voltage clamp at 34 °C. Patch pipette was filled with internal solutions containing (in mM): 138 CsMeSO_4_, 4 NaCl, 10 HEPES, 0.25 EGTA, 10 phosphocreatine, 4 MgATP, and 0.3 NaGTP (295 – 305 mOsm, pH 7.4 with CsOH). For inhibitory measurements, CsMeSO_4_ was substituted for CsCl. For excitatory postsynaptic current (EPSC) and paired pulse ratio (PPR), picrotoxin (50 µM) was added to aCSF. For inhibitory postsynaptic current (IPSC), CNQX (10 µM) and AP5 (50 µM) were added to aCSF. 1 µM of Tetrodotoxin (TTX) was added for all miniature recordings (mEPSC, mIPSC). Pipettes (3–4 MΩ) were pulled from borosilicate glass (Narishige PC-100). Access resistance (Ra) was monitored throughout the recording and cells with >20% increase in Ra were discarded. mEPSCs were analyzed using a template that consisted of an ensemble average EPSCs for each cell, then each event was inspected visually. mIPSCs were analyzed automatically using a detection threshold of 3X root means square (RMS) of noise and inspected visually. Spontaneous excitatory or inhibitory postsynaptic currents (sEPSCs or sIPSCs) were acquired in aCSF without TTX, and 5 min of stable recording was used for analysis. Events were selected automatically using a detection threshold of 3X RMS. Frequency was calculated as number of events/number of seconds recorded. For amplitude, rise time, and decay tau, events were visually inspected and selected based on the rise and decay phases. Cells with <10 clean sEPSCs (<50 for sIPSCs) were excluded from the analysis. Amplitude was detected from individual clean events; rise time and decay tau (mono- or bi-exponential fit; see Supplementary Data for functions used) were calculated from an ensembled average trace per cell [39, 40].

For experiments requiring stimulation, a bipolar tungsten electrode was placed 150 – 200 µm from the recorded cell in the stratum radiatum layer of CA1. Pulse duration for all evoked measures ranged from 40–100 μs and max stimulation intensity <200 µA. PPR recordings were measured with an inter-stimulus interval of 100 ms (10 Hz) every 30 s. First EPSC or IPSC was set to ~200 pA. The ratio was determined as the amplitude of the second response/ the first response. SKF97541 (20 μM) wash-in experiments were performed as previously described [8]. AMPA/NMDA ratio was measured by evoking max EPSCs at a holding potential of -70 mV and +40 mV and analyzed as previously described [41], and the arrow in Fig. 5l denotes the NMDA component.

### Induction of seizures by PTZ

Male and female mice (P65-70) (WT *n* = 7, cKO *n* = 9) were injected with pentylenetetrazol (PTZ) intraperitoneally (i.p.) (10 mg/kg) every 10 min [42] until mice reached a Racine stage (RS) 5 or died. Mice were video monitored in plexiglass chambers. Seizure intensity were quantified according to the modified RS [43]. Briefly, 0 = normal; 1 = sudden behavioral change including motionless staring and orofacial automatisms; 2 = head nodding; 3 = postural changes with forelimb clonus; 4 = rearing and falling with forelimb clonus; 5 = generalized seizure, wild running, and jumping.

### Electroencephalography (EEG) recordings and analysis

Male and female mice (P90-180) (WT *n* = 8, cKO *n* = 10) were anesthetized with a cocktail of ketamine and xylazine (10 mg/kg and 1 mg/kg, i.p.) and implanted with EEG head-mounts (Pinnacle Technologies) containing 2 EEG (Electroencephalography) and 1 EMG (Electromyography) electrodes. EMG wires were inserted into the trapezius muscles. Recording electrodes were then cemented using dental acrylic and the incision was sealed with Vetbond. After 7 days of recovery, each mouse was monitored in a cylindrical plexiglass chamber for continuous chronic tethered EEG/EMG and video recordings for 24 h/day for 14–30 days. Local field potential recordings were acquired at 2 kHz using an EEG monitoring system (SIRENIA® ACQUISITION, Pinnacle Technology) and EEG traces were visually inspected for seizures by blinded investigators with SIRENIA® SEIZURE, as previously described [44, 45].

### Statistical analysis

Inter-group comparisons were conducted using two-tailed Student’s *t*-test (parametric) for data that assumed normal distribution with similar variances between groups or Mann–Whitney test (non-parametric) for data that did not assume normal distribution. Multiple group comparisons were conducted using repeated measures (RM) two-way ANOVA with Sidak’s *post hoc* test. Mixed-effects model was used for multi-group comparisons in which values were missing. Dependent variables are listed in Supplementary Data and genotype was the independent variable for all ANOVAs. Fisher’s exact test was used to compare seizure activity. Sexes were separated for behavioral experiments (three-chamber social approach and nestlet shredding) that reported sex differences. Sexes were combined for remaining behavioral experiments that did not report sex differences. For behavioral analyses, mice were excluded for engaging in behaviors that were not task-related (e.g., jumping, digging, climbing, falling). These non-task specific behaviors were excluded from quantitative analyses according to the criteria of each respective task. For nestlet shredding analyses, animals were excluded if negative values for % nestlet shredded were reported. All data are expressed as mean ± standard error of the mean (SEM).

Number of samples/mouse are indicated inside bars or by their respective plots. Significance was set at *p* < 0.05. All data were analyzed by GraphPad Prism software.

## Results

### *Emx1Cre* driven deletion of *Nrxn2*

We crossed *Nrxn2*^*f/f*^ mice with an *Emx1*^*cre/cre*^ driver to produce *Emx1*-specific *Nrxn2* knockout (*Nrxn2* cKO) mice (Fig. 1a, b). The *Emx1Cre* driver line directs expression of Cre recombinase predominately (~88%) in the excitatory neurons of the neocortex and hippocampus, and also in other cell types and brain regions (Supplementary Fig. 1, [23–26]). Upon Cre recombination in *Emx1* expressing cells, the flanked exon 18-- the first exon shared by *Nrxn2α* and *Nrxn2β*-- is excised leading to the deletion of all reading frames of both *Nrxn2α* and *Nrxn2β*. This conditional mouse model has been validated by Lin et al. [22] wherein excision of exon 18 leads to 99% reduction in *Nrxn2* mRNA levels and the remaining 1% of mRNA was likely detected due to background qRT-PCR measurements. This mouse model has been shown to result in no *Nrxn2α* and *Nrxn2β* mRNA transcripts upon Cre recombination.

We found no significant changes in body or brain weight between WT and *Nrxn2* cKO mice (Supplementary Figure 1). qRT-PCR analyses in different brain regions of WT and *Nrxn2* cKO mice showed a significant reduction in *Nrxn2* expression in the neocortex (*p* < 0.001) and hippocampus (*p* < 0.01), but not cerebellum, of *Nrxn2* cKO mice (Fig. 1c), consistent with the known expression of *Emx1Cre* [23]. The remaining expression observed in *Nrxn2* cKO may be due to the expression of *Nrxn2* in other cell types expressing *Nrxn2* (e.g., GABAergic inhibitory neurons). We saw no differences in *Nrxn1* or *Nrxn3* expression between WT and *Nrxn2* cKO mice (Fig. 1c) confirming the deletion is selective to *Nrxn2*.

### *Nrxn2* cKO mice exhibited sex-dependent preferences for social and non-social cues in a three-chamber social approach

**Fig. 2:**
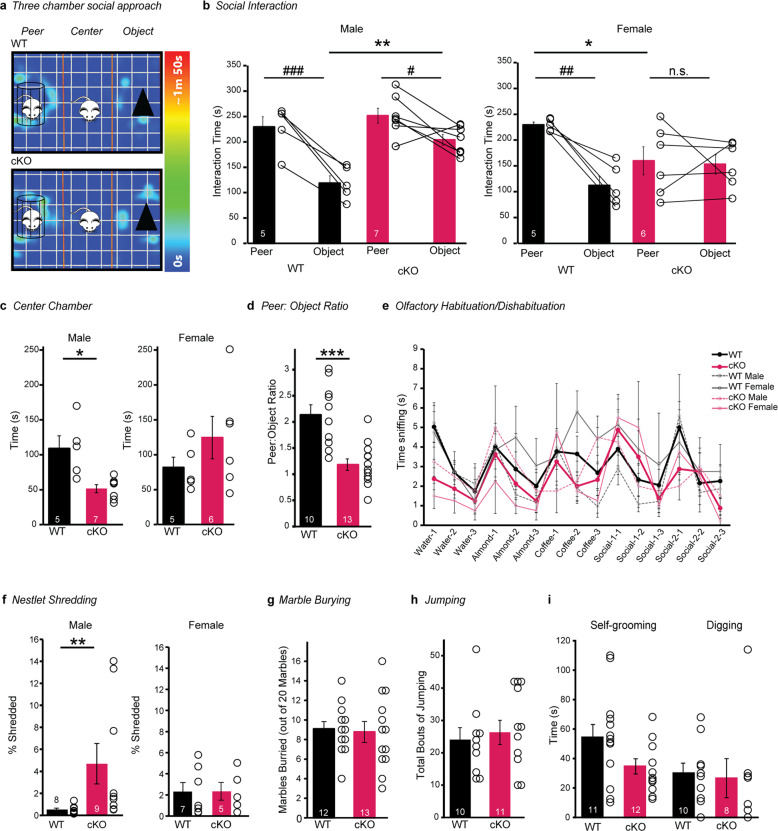
*Nrxn2* cKO mice exhibited a sex-dependent preference for social cues and increased nestlet shredding behavior. **a**–**e** *Nrxn2* cKO mice exhibited a sex-dependent preference for social cues in the three-chamber social approach test with intact olfactory function. **a** Representative heat maps of the three-chamber social approach test. **b** Summary graphs of time spent with peers and objects. **c** Summary graphs of time spent in the center chamber. **d** Summary graph of peer: object ratio. **e** Summary graph depicting time spent sniffing water, non-social odors (almond extract and coffee), and social odors in the olfactory habituation/dishabituation test. **f**–**i** *Nrxn2* cKO males, and not females, exhibited increased nestlet shredding behavior. Both sexes exhibited no changes in other repetitive behaviors measured. **f** Summary graphs of % nestlet shredded. **g**–**i** Summary graphs of number of marbles buried (**g**), total bouts of jumping (**h**), and time spent self-grooming and digging (**i**). Number of mice is listed inside the bar graphs. Each open circle in the summary graphs represents each mouse. Data is represented as means ± SEM from 5 male WT, 7 male *Nrxn2* cKO, 5 female WT and 6 female *Nrxn2* cKO mice for (**b**, **c**); 10 WT and 13 *Nrxn2* cKO for (**d**); 4 male WT and 4 *Nrxn2* cKO, 5 female WT and 4 female *Nrxn2* cKO for (**e**). 8 male WT, 9 male *Nrxn2* cKO, 7 female WT and 5 female *Nrxn2* cKO for (**f**); 12 WT and 13 *Nrxn2* cKO for (**g**); 10 WT and 11 *Nrxn2* cKO for (**h**); 11 WT and 12 *Nrxn2* cKO for self-grooming (**i**); 10 WT and 8 *Nrxn2* cKO for digging (**i**). Data is collected from two independent cohorts of mice for panels (**f**, **g**) and (**h**, **i**). Statistical assessments were performed by RM two-way ANOVA followed by Sidak’s *post hoc* test (**b**, **e**) or two-tailed Student’s *t*-test (**c**, **d**, **g**–**i**) or Mann–Whitney test (**f**) by comparing *Nrxn2* cKO to WT with n.s. = not significant *p < 0.05, **p < 0.01, ***p < 0.001, ****p < 0.0001 and by comparing interaction time with peer and object within each genotype with ^#^*p* < 0.05, ^##^*p* < 0.01, ^###^*p* < 0.001.

We first assessed social behaviors since the hippocampus and cortex are implicated in social behavior [46–51]. In male WT and *Nrxn2* cKO mice, RM two-way ANOVA showed a significant effect of cue [F(1,10) = 33.33; *p* < 0.001] and genotype [F(1,10) = 10.22; *p* < 0.01] on interaction time with significant cue x genotype interaction [F (1,10) = 5.301; *p* < 0.05]. *Post hoc* analyses showed both WT and *Nrxn2* cKO males (WT: *t* = 5.287, *p* < 0.001; cKO: *t* = 2.689; *p* < 0.05) exhibited social preference. Further, *post hoc* analyses confirmed that male *Nrxn2* cKO mice exhibited an increased preference for non-social cues than WT mice (*t* = 3.927, *p* < 0.01) (Fig. 2b). In female WT and *Nrxn2* cKO mice, RM two-way ANOVA showed a significant effect of cue [F (1,9) = 11.26; *p* < 0.01], but not genotype, on interaction time along with a significant cue x genotype interaction [F (1,9) = 8.910; *p* < 0.01]. *Post hoc* analyses confirmed that female WT (*t* = 4.293; *p* < 0.01), but not *Nrxn2* cKO (*t* = 0.2749; *p* = 0.9557), spent more time interacting with peers (Fig. 2b). Further, *post hoc* analysis confirmed that female *Nrxn2* cKO mice spent less time interacting with social cue than female WT mice (*t* = 2.459; *p* < 0.05) indicating social deficits (Fig. 2b)

Male *Nrxn2* cKO mice, but not female *Nrxn2* cKO mice, spent less time in the middle chamber in comparison to WT mice (*p* < 0.05; Fig. 2c). Lastly, these observations in both male (increased object interaction time) and female (decreased peer interaction time) *Nrxn2* cKO mice are further demonstrated by a lower peer/object ratio (*P* < 0.001; Fig. 2d).

We next examined if social impairments observed in *Nrxn2* cKO are contributed by altered olfactory function because *Emx1* also expresses in olfactory bulb GABAergic interneurons [24]. There was a significant effect of the trial on sniffing time for water [F (1.627, 24.25) = 7.424; *p* < 0.01], almond extract [F (1.501, 22.51) = 6.560; *p* < 0.01], social cue-1 [F (1.921, 28.81) = 6.509; *p* < 0.01], social cue-2 [F (1.622, 24.33) = 3.661; *p* < 0.05], but not for coffee, indicating olfactory habituation upon repeated exposure to social and non-social odors in both WT and *Nrxn2* cKO mice. Except for water [F (1,15) = 5.325; *p* < 0.05], there was no significant effect of genotype on sniffing time for almond extract, coffee, and both social cues. Results from the olfactory habituation/dishabituation test showed that the sense of smell is intact in *Nrxn2* cKO mice (Fig. 2e).

### Male *Nrxn2* cKO mice exhibited increased nestlet shredding behavior

We investigated repetitive behaviors by monitoring nestlet shredding, marble burying, jumping, digging, and self-grooming behaviors in *Nrxn2* cKO. Male *Nrxn2* cKO mice, but not female *Nrxn2* cKO mice, exhibited a significant increase % of nestlet shredded compared to their WT littermates (Male *Nrxn2* cKO v/s male WT: *p* < 0.01; Fig. 2f). There were no significant differences in the number of marbles buried between the WT and *Nrxn2* cKO mice (Fig. 2g). Finally, we did not observe any significant differences in bouts of jumping (Fig. 2h) or time spent in grooming and digging behaviors (Fig. 2i) between WT and *Nrxn2* cKO mice.

### General activity and memory in *Nrxn2* cKO mice remain intact

**Fig. 3:**
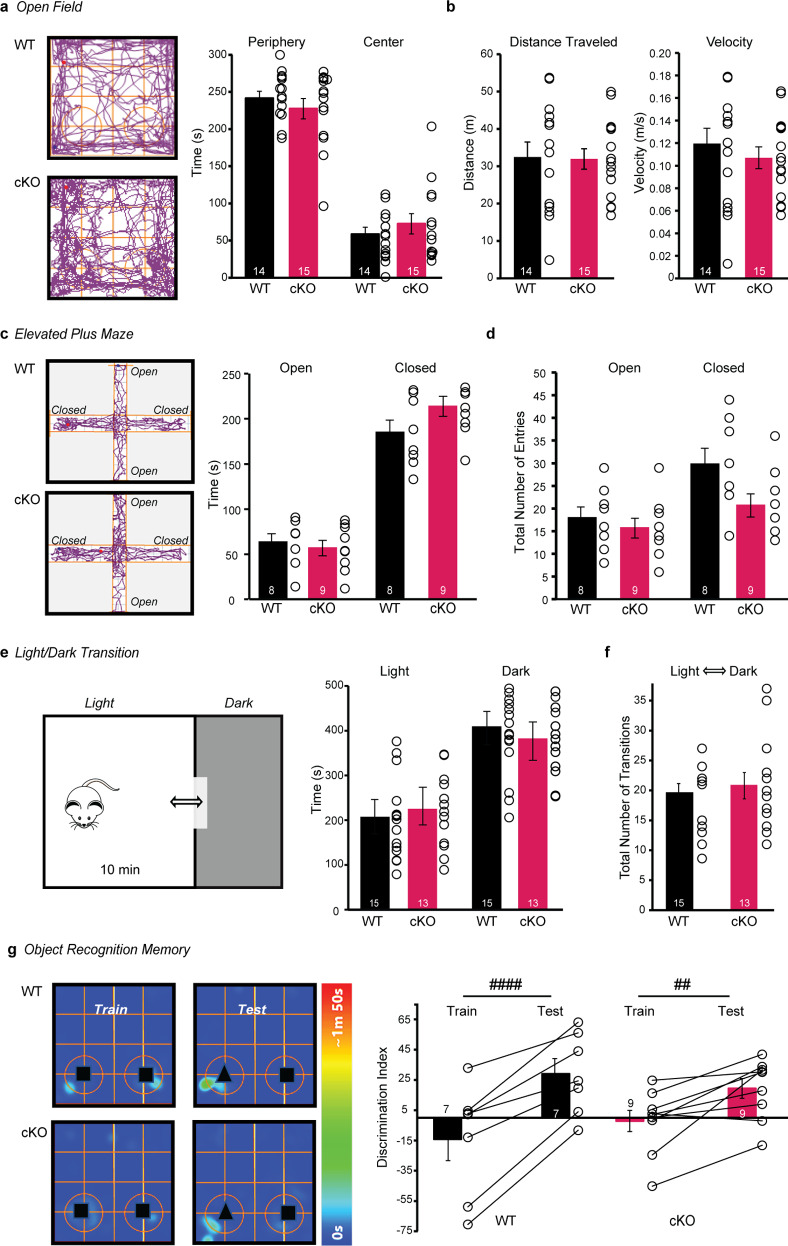
General activity and memory in *Nrxn2* cKO mice remained intact. **a**, **b** *Nrxn2* cKO mice exhibited normal general mobility without alterations in anxiety behavior in open field test. **a** Representative track plot of distance traveled in the open field (left); summary graph of time spent in the periphery and center in open field test (right), **b** distance traveled (left) and velocity(right). **c–f** *Nrxn2* cKO mice did not exhibit anxiety-like behavior in EPM and L/D transition tests. **c** Representative track plot from EPM test (left); summary graph of time spent in open and closed arms of elevated plus maze test (right). **d** Summary graph of total number of entries in the open and closed arms of elevated plus maze. **e** Representative image of light/dark transition test (left); summary graph of time spent in the light and dark compartments in the light/dark transition test(right). **f** Summary graph of total number of transitions between light and dark compartments in the light/dark transition test. **g** *Nrxn2* cKO mice did not exhibit working memory deficits in the object recognition memory test. Representative heat maps of object recognition memory (left); summary graph of discrimination index during training and testing sessions (right). Number of mice is listed inside the bar graphs. Each open circle in the summary graphs represents each mouse. Data is represented as means ± SEM from 14 WT and 15 *Nrxn2* cKO for time spent in periphery and center (**a**), distance traveled and velocity (**b**); 8 WT and 9 *Nrxn2* cKO for (**c**, **d**); 15 WT and 13 *Nrxn2* cKO for (**e**, **f**); 7 WT and 9 *Nrxn2* cKO for (**g**). Statistical assessments were performed by two-tailed Student’s *t*-test (**a–f**), or RM two-way ANOVA followed by Sidak’s *post hoc* test (**g**) by comparing training to test sessions within each genotype with ^####^*p* < 0.0001, ^##^*p* < 0.01.

We next analyzed the effects of *Nrxn2* deletion on anxiety and cognitive behaviors. In the open field test, there were no significant differences in the duration spent in the periphery and center, distance traveled, and velocity between WT and *Nrxn2* cKO mice (Fig. 3a, b). In the elevated plus maze test, there were no significant differences between WT and *Nrxn2* cKO mice in the time spent in the open or closed arms (Fig. 3c) or in the number of open arm entries (Fig. 3d), but a non-significant trend towards decreased closed arm entries in *Nrxn2* cKO mice (*p* = 0.0508; Fig. 3d). In light/dark transition test, there were no significant differences between the WT and *Nrxn2* cKO mice in the time spent in the light or dark compartments (Fig. 3e). No significant differences were observed in the number of transitions between the light and dark compartments. (Fig. 3f).

To evaluate memory, we performed an object recognition memory test which is known to involve the hippocampus and other cortical areas [52]. Two-way ANOVA showed a significant effect of session [F (1,14) = 50.64; *p* < 0.0001], but not genotype on discrimination index along with a significant genotype x session interaction [F (1,14) = 5.596; *p* < 0.05]. *Post hoc* analyses confirmed that WT (*t* = 6.321; *p* < 0.0001) and *Nrxn2* cKO (*t* = 3.591; *p* < 0.01) (Fig. 3g) mice exhibited a significantly higher discrimination index in the test session than in the training session indicating that *Nrxn2* deletion did not affect memory.

### *Nrxn2* cKO mice did not exhibit any changes in cortical layer thickness

**Fig. 4:**
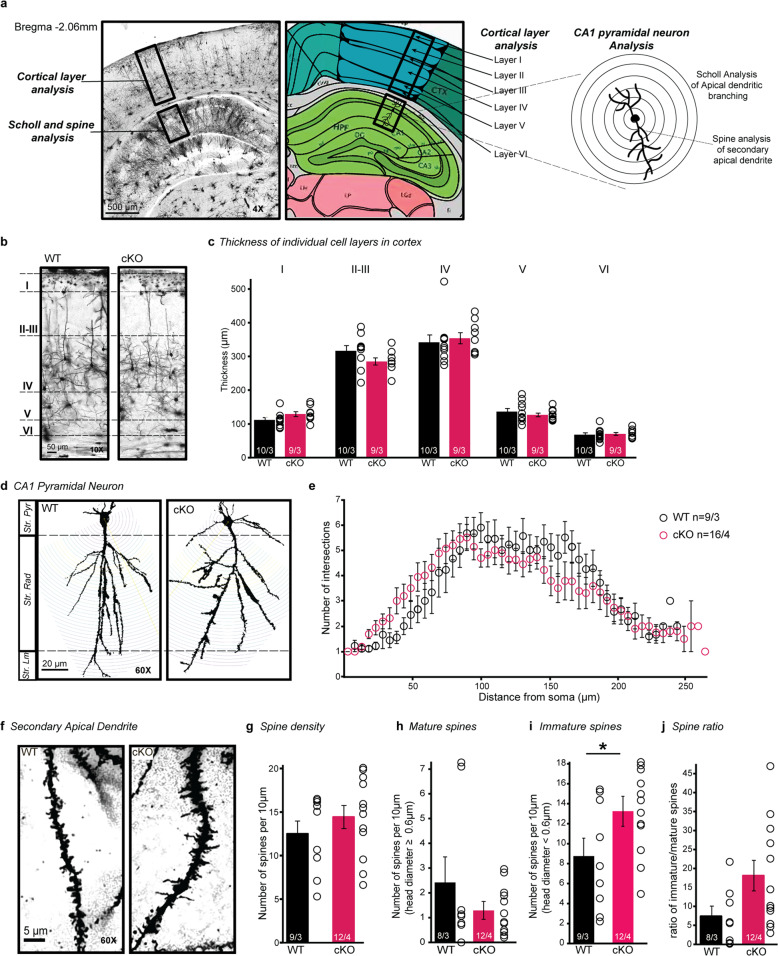
Increased immature spines in hippocampal CA1 of *Nrxn2* cKO mice. **a** Overview scheme to illustrate the selected area and analysis criteria for cortical layers (**b**, **c**), dendritic branching (**d**, **e**), and dendritic spine (**f**–**j**). Brightfield image (left) of a Golgi-stained brain tissue. Labeled boxes show cortical and dorsal hippocampal regions selected for analysis. Image adapted from Allen brain atlas (right) demonstrates relative thickness of individual cortical layers at Bregma −2.06 mm. **b**, **c** Conditional deletion of *Nrxn2* did not change the thickness of cortical cell layers. **b** Representative image (10X) of Golgi-stained cortex of WT and *Nrxn2* cKO. **c** Summary graph of thickness for each layer. **d**, **e**. Conditional deletion of *Nrxn2* did not alter dendritic arborization of the CA1 pyramidal neurons in dorsal hippocampus. **d** Images of CA1 pyramidal neuron apical dendrites. **e** Summary graph depicting the number of intersections in CA1 pyramidal neurons dendrite using Sholl analysis. **f–j** Conditional deletion of *Nrxn2* increased total number of immature spines without changes in spine density or spine ratio in the stratum radiatum (Str. Rad) CA1 region. **f** Magnified representative images of secondary apical dendritic segments. **g** Summary graph of spine density per 10 µm. **h**–**j** summary graphs of number of mature (**h**) and immature (**i**) spines per 10 µm. **j** Summary graph of ratio of immature spines to mature spines. Number of sections analyzed/mouse (**c**), pyramidal neurons/ mouse (**e**), or dendrites/mouse (**g–j**) is listed inside the bar graphs. Data is represented as ±SEM from 3 WT and 4 *Nrxn2* cKO mice. Statistical assessments were performed by two-tailed Student’s *t*-test (**c**, **g–i**) or two-way ANOVA (**e**) by comparing *Nrxn2* cKO to WT with *p < 0.05.

*Nrxn2* cKO mice did not exhibit any differences in cortical layer thickness in comparison to WT mice. However, the difference in cortical layer thickness between WT and *Nrxn2* cKO mice was trending towards statistical significance in layer I (*p* = 0.09145). (Fig. 4b).

### Increased immature spines in hippocampal CA1 of *Nrxn2* cKO mice

*Nrxn2* encodes a cell-adhesion molecule located at the synapse [11], and abnormalities in dendritic spine density, arborization, and maturity are key hallmarks of ASD [53–56]. To test if *Nrxn2* deletion disrupted spine morphology, we performed Golgi analysis on WT and *Nrxn2* cKO mice. Two-way ANOVA analysis showed no significant effect of genotype on dendritic arborization [F (1,5) = 1.082; *p* = 0.3459; Fig. 4e], indicating normal branching of dendritic trees in CA1 region in *Nrxn2* cKO. Moreover, there was no significant difference in spine density in CA1 stratum radiatum between genotypes (*p* = 0.3444; Fig. 4g).

Next, we examined the dendritic spine morphology by measuring the spine head diameter and found no difference in the density of mature spines between WT and cKO mice (*p* = 0.807; Fig. 4h). However, *Nrxn2* cKO mice exhibited a significantly higher number of immature spines compared to WT littermates (*p* < 0.05; Fig. 4i). The greater number of immature spines in *Nrxn2* cKOs was also reflected in a non-significant trend towards an increased ratio of immature spines to mature spines between WT and *Nrxn2* cKO mice (*p* = 0.0644; Fig. 4j).

### Conditional deletion of *Nrxn2* altered synaptic transmission in CA1

**Fig. 5:**
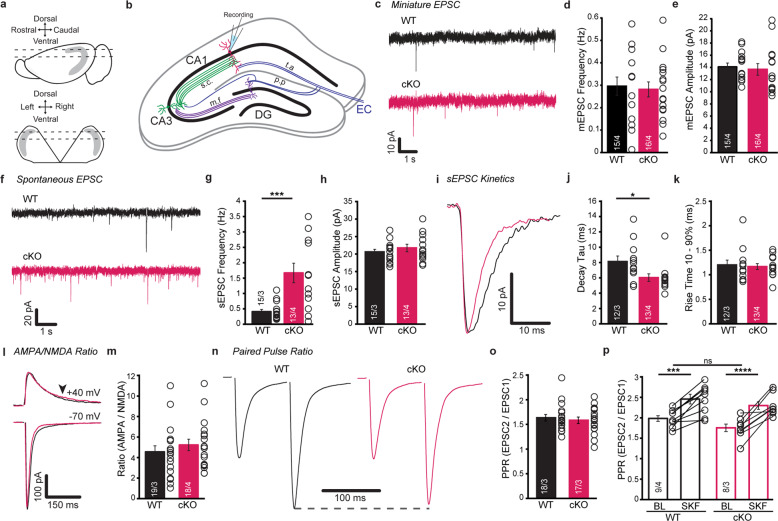
Conditional deletion of *Nrxn2* increases network activity in the CA3-CA1 circuit. **a**, **b** CA1 pyramidal cells were recorded from acute semi-horizontal slices. Illustrations show semi-horizontal hippocampal slices prepared from a 12-degree angle (**a**) and inputs are preserved in slices (**b**). Dashed lines denote the range of slices collected for recording. **c**–**e** Conditional deletion of *Nrxn2* does not impact basal excitatory synaptic transmission. **c** Representative mEPSC traces. Summary graphs of frequency (**d**) and amplitude (**e**). **f**–**h** Conditional deletion of *Nrxn2* increases the frequency of sEPSCs. **f** Representative sEPSC traces. Summary graphs of frequency (**g**) and amplitude (**h**). **i**–**k** Conditional deletion of *Nrxn2* decreases the decay time constant (tau) of sEPSCs. **i** Representative traces of averaged sEPSC event. Summary graphs of decay tau (**j**) and rise time (**k**). **l**, **m** Conditional deletion of *Nrxn2* does not impact the ratio of AMPA- and NMDA-mediated currents. **l** Representative traces of evoked EPSCs. **n** Summary graph of AMPA/NMDA ratio. **n**, **o** Conditional deletion of *Nrxn2* does not impact the release probability of glutamate, assessed by measuring paired-pulse ratio (PPR). **n** Representative traces of excitatory PPR. **o** Summary graph of PPR. **p** Conditional deletion of *Nrxn2* does not impact GABA_B_-mediated function on glutamate release probability. Summary graph of PPR before (baseline, BL) and after application of SKF (SKF97541, 20 µM). Number of neurons/mice is listed inside the bar graphs. Open circles represent each cell. Data is represented as means ± SEM for 4 WT and 4 *Nrxn2* cKO for (**d**, **e**); 3 WT and 3 *Nrxn2* cKO for (**g**, **h, j**, **k**, **m**); 3 WT and 3 *Nrxn2* cKO for (**o**); 4 WT and 3 *Nrxn2* cKO (**p**). Statistical assessments performed by unpaired two-tailed Student’s *t*-test by comparing *Nrxn2* cKO to WT mice with *p < 0.05, ***p < 0.001,****p < 0.0001.

Although we did not observe a change in spine density in *Nrxn2* cKO, we examined if the significant increase in immature spines could alter CA1 synaptic transmission by recording mEPSCs from CA1 pyramidal neurons in WT and *Nrxn2* cKO mice. No changes were observed in mEPSC frequency or amplitude between WT and *Nrxn2* cKO mice (Fig. 5c–e). Next, we measured sEPSC to examine the role of *Nrxn2* on hippocampal network activity. Compared to WT mice, CA1 pyramidal neurons in *Nrxn2* cKO mice showed a 4-fold increase in the frequency of sEPSCs (*p* < 0.01) but no change in the amplitude of sEPSCs (Fig. 5f–h). Kinetics analyses showed a significant decrease in the decay time constant (*p* < 0.05), but no change in 10-90% rise time was detected (Fig. 5i-k).

The increased frequency of sEPSCs in CA1 pyramidal cells could result from changes to either the pre- or postsynaptic machinery [57]. Therefore, we examined evoked EPSC responses in CA1 to stimuli applied to Schaffer collaterals in the stratum radiatum. To test if *Nrxn2* cKO disrupts postsynaptic transmission, we measured the ratio between evoked AMPA- and NMDA-mediated currents. No differences in AMPA/NMDA ratio were detected (Fig. 5l, m). To test if *Nrxn2* cKO alters presynaptic release probability, we applied paired stimuli and calculated the PPR of evoked EPSCs. No difference in PPR between WT and *Nrxn2* cKO mice was observed (Fig. 5n, o). Finally, a recent report showed that Nrxns regulate GABA_B_ receptor-mediated function [8], we examined the effect of *Nrxn2* cKO on GABA_B_ receptor modulation of excitatory PPRs. In the presence of the potent GABA_B_ receptor agonist, SKF 97541, we detected no differences in GABA_B_ receptor activation on glutamate release probability between WT and cKO mice (Fig. 5p). *Nrxn2* cKO had a similar, albeit smaller, increase in the frequency of spontaneous inhibitory synaptic transmission in CA1 pyramidal neurons and no effect on evoked or basal transmission (Fig. S2). Taken together, our results suggest *Nrxn2* deletion produces an increase in synaptic excitability in hippocampus.

### *Nrxn2* cKO mice had a lower threshold for evoked seizures and developed spontaneous seizures

**Fig. 6:**
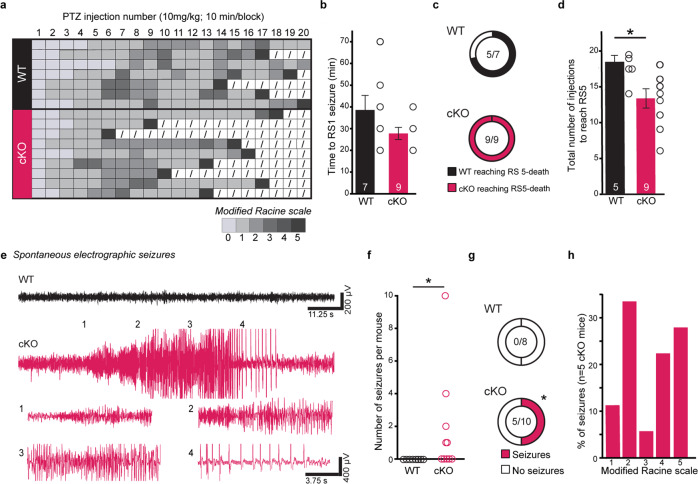
*Nrxn2* cKO mice exhibited higher susceptibility to chemically evoked seizures and develop spontaneous reoccurring behavioral seizures. **a**–**d** *Nrxn2* cKO mice were susceptible to pentylenetetrazol (PTZ)-induced seizures and seizure-related death. **a** Diagram illustrating Racine Stage (RS) response and mortality of WT and *Nrxn2* cKO after PTZ injections. Each row indicates an individual mouse, and columns indicate the injection number. RS response (0–5) is represented by shades of gray. “/” indicate no further injections due to death. **b** The first seizure-related behavior (RS 1) observed, **c** total number of mice that reached RS5 (death) by 20 injections, **d** summary graph of total number of PTZ injections to induce generalized seizures (RS5). **e**–**h** *Nrxn2* cKO mice exhibit unprovoked spontaneously reoccurring seizures. **e** Representative traces of EEG. *Nrxn2* cKO traces show unprovoked electrographic seizures (total seizure duration = 64 s). Insets: Enlarged EEG traces showing classical electrographic seizure characteristics (evolution of EEG activity with >two-fold increase in amplitude and frequency). See Supplemental Video 2 for timestamped behavioral seizures. **f** Summary graph of total number of seizures. **g** Number of mice with spontaneous electrographic seizures (filled) or without spontaneous electrographic seizures (unfilled). **h** Distribution of Racine scale seizure behavior in *Nrxn2* cKO mice with spontaneous electrographic seizures. Number of mice is listed inside the bar graphs. Each open circle represents each mouse. Data is represented as means ± SEM from 7 WT and 9 *Nrxn2* cKO for (**b**); 5 WT and 9 *Nrxn2* cKO for (**d**); 8 WT and 10 *Nrxn2* cKO for (**f**–**h**). Discrepancy in number of WT mice for (**b**) and (**d**) is due to 2 WT mice not reaching RS5 as shown in (**a**). Statistical assessments were performed by two-tailed Student’s *t*-test (**b**, **d**) or Fisher’s exact test (**c**, **f**, **g**) by comparing *Nrxn2* cKO to WT mice with *p < 0.05.

Increased network excitability in vitro could indicate an increased risk for epilepsy in vivo, a phenotype observed in humans with *NRXN2* mutations [14, 16]. To test this possibility, we first examined the threshold for evoked seizures after PTZ administration (Fig. 6a). There was no difference in latency to the first RS1 behavior between genotypes (Fig. 6b). After 20 PTZ injections, all *Nrxn2* cKO mice resulted in RS5 related death, but not all WT mice (Fig. 6c). *Nrxn2* cKO mice required significantly fewer PTZ injections to induce convulsive, RS5 seizure behaviors compared to age-matched WT littermates (*p* < 0.05; Fig. 6d; Supplementary Video 1).

To test for spontaneous electrographic seizures, the hallmark of epilepsy, we performed 2–4 weeks of continuous (24 h/day) video-EEG monitoring in WT and *Nrxn2* cKO mice. Non-fatal, spontaneously reoccurring, electrographic, and behavioral seizures were detected in half of the *Nrxn2* cKO mice (Fig. 6e, g). Seizures consisted of high-frequency, high-amplitude, rhythmic activity with clear onset and termination (*p* < 0.05; Fig. 6e–g). Simultaneous video monitoring confirmed a mixture of convulsive (RS3-5) and non-convulsive (RS1-2) seizure behaviors during electrographic events (Fig. 6h, Supplemental Video 2). Electrographic or behavioral seizures were never observed in WT mice (Fig. 6e–g).

## Discussion

Our findings have identified a novel circuit-specific role for *Nrxn2* in increased network activity in the hippocampal circuitries, and the manifestation of associated behavioral phenotypes in *Nrxn2* cKO mice. Further, we have observed spontaneously reoccurring electrographic and behavioral seizures in *Nrxn2* cKO mice that have not been previously demonstrated in other *Nrxn* mutant mouse models.

Female *Nrxn2* cKO mice displayed social deficits as evidenced by a lack of social cue preference. In contrast, male *Nrxn2* cKO mice were more exploratory in their surroundings as indicated by their increased preference for non-social cues and decreased latency to enter the side chambers. Further, increased nestlet shredding behavior was found only in male *Nrxn2* cKO mice. The sex differences in our behavioral data align with the previous finding from germline *Nrxn2* KO [17] suggesting a sex-dependent role for *Nrxn2* in social and repetitive behaviors. The lack of social reward experience and the repetitive shredding behavior in *Nrxn2* cKO mice could be a consequence of a disrupted connection from the prefrontal cortex to the dorsal striatum, part of the basal ganglia circuitry which underlies behavioral flexibility and reward processing [58–60]. Future investigations on the synaptic function and behaviors mediated by the corticostriatal circuits would expand our current understanding of the role of Nrxn2.

To gain insights into cellular and synaptic mechanisms underlying behavioral deficits in *Nrxn2* cKO mice, we assessed the effects of *Nrxn2* deletion on the development of cortical layer, dendritic branching, dendritic spine morphology, and hippocampal network activity. Our *Nrxn2* cKO data showed a significant increase in the number of immature spines, with no changes in mature spines or dendritic branching in the hippocampus or cellular layers in the cortex. Although overall spine density remained unchanged in *Nrxn2* cKO mice, the increase in immature spines presented the possibility that *Nrxn2* cKO resulted in additional synapses being formed. Our mEPSCs results showed that the increased immature spine density may not be functional or does not influence overall synaptic function. Studies have shown that the morphological changes of dendritic spines (e.g., enlargement of the dendritic spine head) are essential for long-term potentiation and memory consolidation, which allows for AMPAR insertions in response to stimulations (e.g., electrical stimulation in brain slices or through learning) [61, 62]. The small trend towards an increased ratio of immature to mature spines in naïve (unstimulated) *Nrxn2* cKO mice suggests that these naïve immature spines could potentially become mature upon stimulation or learning to form stable synaptic connections. This data corresponds to our observations in electrophysiology recordings and behavioral tasks where naïve *Nrxn2* cKO exhibit abnormal phenotypes but can appear normal upon stimulation or learning.

Increased frequencies of sEPSCs and sIPSCs in *Nrxn2* cKO mice suggest that *Nrxn2* deletion alters basal network activity. These changes were independent of changes in neurotransmitter release probability or changes in the relative contribution of AMPA and NMDA receptors. Further analysis of the tri-synaptic hippocampal circuit by measuring the population field potential (excitatory postsynaptic potential, EPSP) via Schaffer-collateral stimulation evoked EPSP, sharp wave, and high-frequency oscillation in acute hippocampal slices could provide insight into the impact of *Nrxn2* deletion on overall hippocampal network activity [63, 64]. Moreover, a recent study suggested a canonical regulatory role of neurexins on presynaptic GABA_B_ receptor mediated function on glutamatergic transmission and release probability [8]. However, our data showed that *Nrxn2* cKO might not share the canonical role as other *Nrxns*. Further investigations into GABAergic transmissions, such as tonic and feedforward inhibition, would advance our understanding of the role of *Nrxn2* in synaptic transmission and hippocampal circuit function.

*Emx1Cre-*driven deletion *Nrxn2* resulted in abnormal synchronization of neuronal activity in the hippocampal circuitry, as evidenced by the low threshold to PTZ-induced seizures and the occurrence of spontaneous seizures in *Nrxn2* cKO mice. Seizure-inducing drugs and local stimulation such as optogenetics are commonly used to evaluate seizure susceptibility [65–68]; however, these induction paradigms do not model *spontaneous* seizures, which is the hallmark of epilepsy. During EEG monitoring, we found non-fatal, unprovoked (spontaneous), behavioral (Racine scaled), seizures (lasting >30 s) that reoccurred (>once) in ~50% of *Nrxn2* cKO mice. This is an important finding because the presence of spontaneous seizures is not always robust in genetic mouse models of epilepsy [69, 70]. The high susceptibility of *Nrxn2* cKO mice to spontaneously recurring seizures is a critical piece of information since epilepsy has been diagnosed in 9-19% of ASD patients [71]. Furthermore, ASD shares a high degree of comorbidity with syndromic neurodevelopmental disorders (Fragile X, tuberous sclerosis complex, Timothy syndrome, and Phelan-McDermid syndrome) that are also characterized by spontaneously recurring seizures. For instance, 60% of patients diagnosed with Fragile X syndrome meet the diagnostic criteria for ASD [72–74]. 50–60% of children with tuberous sclerosis complex meet the diagnostic criteria for ASD by 3 years of age [75, 76]. Timothy syndrome, a rare multisystem disorder induced by mutations in the *CACNA1C* gene is one of the most penetrant forms of ASD with percentages as high as 60–80% [77]. 26–84% of patients diagnosed with Phelan-McDermid syndrome exhibit ASD-like behavioral traits, demonstrating ASD as the common comorbidity [78, 79]. Considering that 14% of all *NRXN2* mutations reported have resulted in both ASD and ASD-associated disorders (Supplemental Table 1), the *Nrxn2* cKO mouse model is a valuable genetic tool to study biological pathways underlying the co-morbidity of ASD with epilepsy and other neurodevelopmental disorders [80]. Overall, our findings will be a stepping stone toward establishing a role for *Nrxn2* in the pathogenesis of disorders characterized by hyperexcitability and altered social behaviors including ASD and co-morbid neurological and developmental disorders.

## Supplementary information

Supplementary video 1

Supplementary video 2

Supplementary Figure 1

Supplementary Figure 2

Supplementary Table 1

Supplementary Table 2

Supplementary Data
