# Supplementary figures and images for: Conditional deletion of Neurexin-2 alters neuronal network activity in hippocampal circuitries and leads to spontaneous seizures
Source: Transl Psychiatry. 2023 Mar 20;13:97. doi: 10.1038/s41398-023-02394-6 (PMC10027846; doi:10.1038/s41398-023-02394-6)

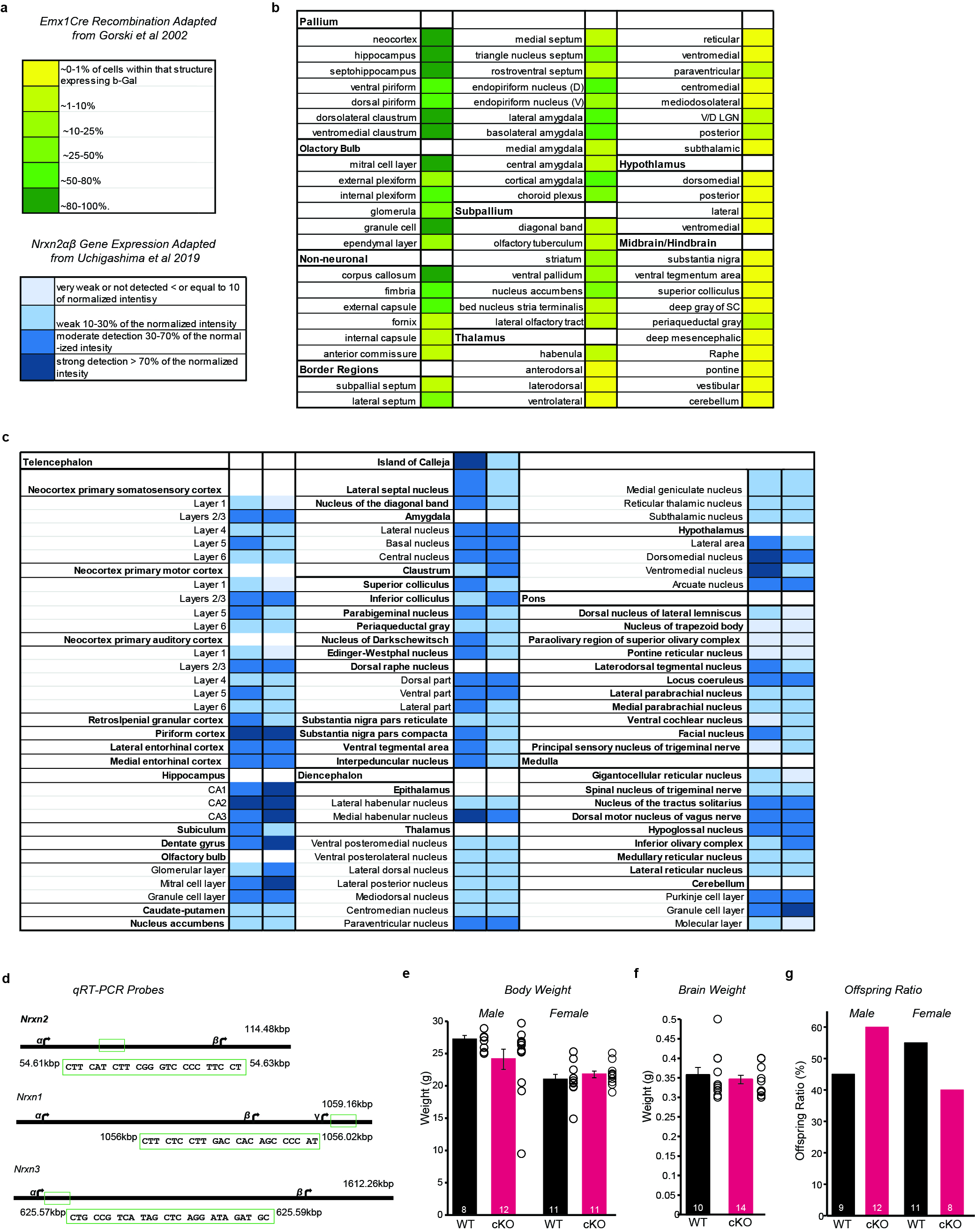

Supplement: Supplementary file 3 — Supplementary Figure 1 [file 41398_2023_2394_MOESM3_ESM.jpg]

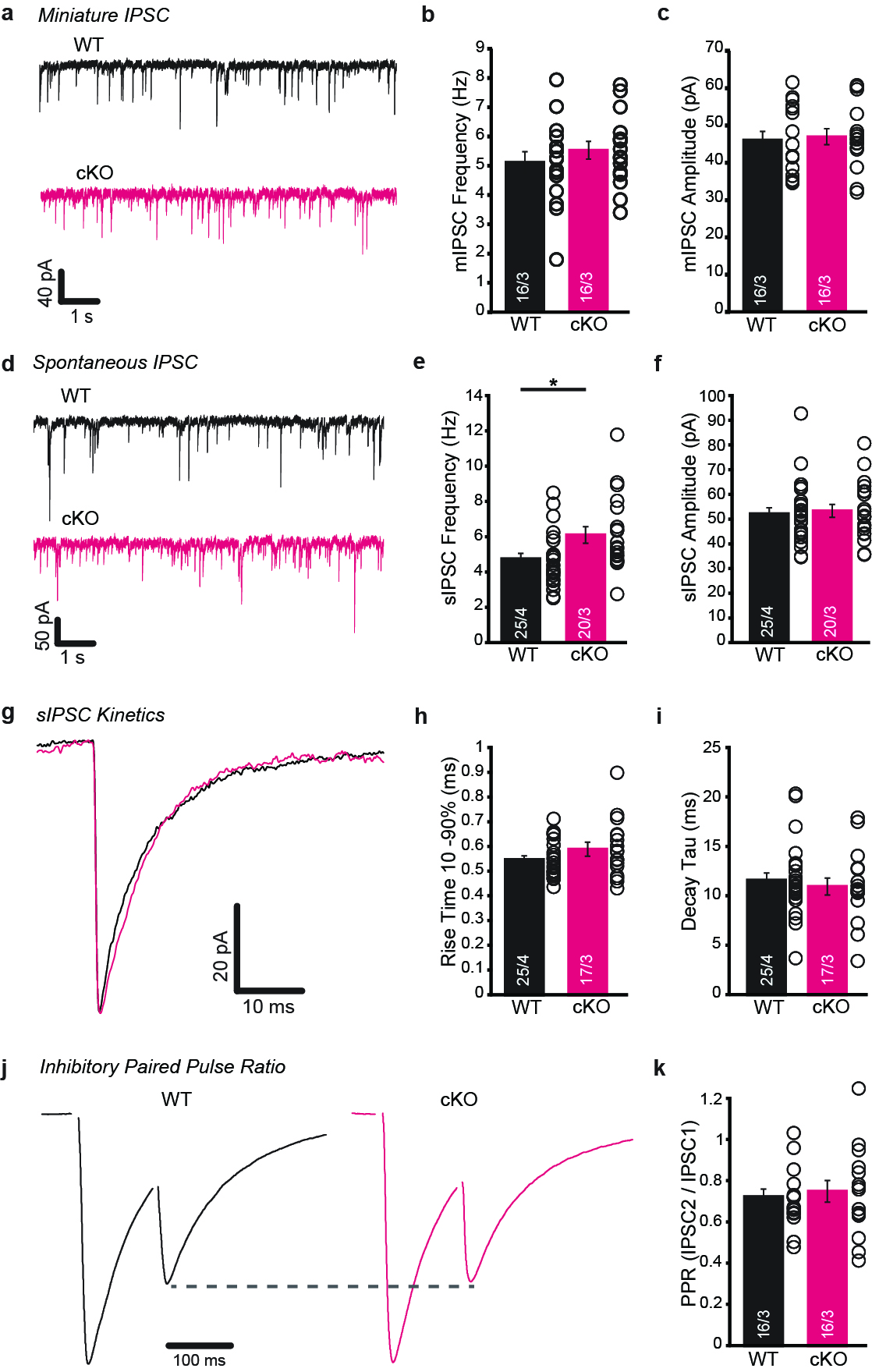

Supplement: Supplementary file 4 — Supplementary Figure 2 [file 41398_2023_2394_MOESM4_ESM.jpg]
